# Magnitude and Predictors of Dietary Diversity among HIV-Infected Adults on Antiretroviral Therapy: The Case of North-Western, Ghana

**DOI:** 10.1155/2024/2777908

**Published:** 2024-01-30

**Authors:** Louis Nebayeng Mornah, Mahama Saaka, Diana Pireh

**Affiliations:** ^1^University for Development Studies, Department of Nutritional Sciences, Tamale, Ghana; ^2^Ghana College of Nurses and Midwives, Pediatric Faculty, Tamale, Ghana

## Abstract

**Introduction:**

Though people living with HIV/AIDS require a good combination of antiretroviral therapy and healthy dietary habits for a quality life and positive medical outcomes, little is, however, known regarding the dietary practices of HIV-positive patients who receive antiretroviral therapy (ART) in the Lawra Municipality.

**Objective:**

This study assessed the magnitude and factors associated with dietary diversity among HIV-positive patients on antiretroviral therapy (ART).

**Methods:**

This study was a facility-based cross-sectional study of 269 study participants recruited using a systematic random sampling technique. Bivariate and multivariable logistic regression analyses were performed to identify factors associated with their dietary diversity.

**Results:**

This study shows that only 36 (13.4%) of the sample consumed a diversified diet with a mean dietary diversity score of 3.7 ± 0.99. Starchy staple foods (96.7%) and flesh food (92.9%) were the most consumed foods. Being a nonfarmer employee (AOR = 10.76, 95% CI = 1.03–112.35), not taking cotrimoxazole prophylaxis (AOR = 3.76, 95% CI = 1.02–14.37) and adults of age 18–27 years (AOR = 5.95, 95% CI = 1.18–30.07) were significant predictors of high dietary diversity.

**Conclusion:**

This study revealed that dietary diversity was a significant nutritional problem among HIV-positive adults in Lawra Municipal Hospital. Starchy staple foods and flesh food were the most consumed foods, while organ meats, dairy products, and eggs were eaten less. Having a secured salary paid job, not taking cotrimoxazole prophylaxis, and being a young adult were strong predictors of a high dietary diversity score. Therefore, efforts should be made to strengthen and improve the economic status and to educate these vulnerable groups on the need to adhere to cotrimoxazole prophylaxis uptake.

## 1. Introduction

Effective dietary management is a promising activity in the treatment of human immunodeficiency virus (HIV) and/or acquired immunodeficiency syndrome (AIDS) [1]. Therefore, a diversified diet is required to ensure that HIV-/AIDS-affected persons can work and contribute to their household's and country's socioeconomic growth [2, 3]. Dietary diversity is a quantifiable amount of food groups that are often used to determine the variety and nutritional adequacy of diets [4]. These diversified diets may include food from either vegetables, fruits, grains, or animal sources. Their main role is to promote healthy growth and development as well as boost the immunity of the patient [5].

The consumption of an adequate balanced diet increases resistance to infection and disease as well as promotes good health and increases the productivity of people living with HIV/AIDS (PLWHA). A well-diversified diet improves energy and builds the person's immune system in the fight against opportunistic infections and other complications. This state of health is also required for the treatment of malnutrition specifically undernutrition among HIV patients [6]. Inadequate dietary intake can greatly contribute to poor nutritional status and subsequently result in low immune functions [6]. The consumption of an adequate balanced diet increases resistance to infection and disease, promotes good health, and increases the productivity of PLWHA. A well-diversified diet improves energy and builds the person's immune system in the fight against opportunistic infections and the treatment of undernutrition among HIV patients [6].

Globally, an undiversified diet among persons living with HIV/AIDS is a public health concern, particularly in poorer nations [7]. HIV-infected patients with opportunistic infections are at high risk of poor nutritional status due to the intake of a low-quality, monotonous diet, which leads to micronutrient and macronutrient deficiencies [3].

The Ghana Ministry of Health has taken great steps in addressing nutrition among People Living with HIV (PLHIV) by launching the Nutritional Assessment, Counseling, and Support program [8]. Dietary variety among HIV-positive individuals 15 years and above is impacted by numerous factors. This includes reduced appetite, low educational level and poor socioeconomic status, availability of nutritional counseling sessions, and higher disease progression resulting in micronutrient deficiencies [9]. Available evidence suggests that people living with HIV/AIDS require a good combination of antiretroviral therapy and healthy dietary habits for a quality life and positive medical outcomes [10].

However, limited data are available regarding the dietary practices of HIV-positive patients receiving antiretroviral therapy (ART) in the Lawra municipality of Northern Ghana, since there are few works on dietary practices, i.e., nutritional status assessment of HIV-positive patients receiving antiretroviral therapy in Ghana. More so, those studies were conducted in cosmopolitan cities [11] and hence accounted for diverse foods across many geographical areas in Ghana. Therefore, this study assessed the magnitude and factors associated with dietary diversity practices among HIV-positive patients attending antiretroviral therapy (ART).

## 2. Methods and Materials

### 2.1. Study Setting

This study was conducted in Lawra, Ghana. Lawra is the capital of the Lawra Municipality and one of the oldest administrative districts of the Upper West Region (Lawra Municipal Health Administration, 2018, unpublished). The municipality has only one antiretroviral therapy (ART) site located in the Municipal Hospital and provides voluntary counseling and testing (VCT) services to both locals and the neighboring country Burkina Faso.

### 2.2. Study Design and Sampling

This study was a facility-based cross-sectional study. The target population was all adults (15 years and above) who were infected with HIV/AIDS, living in Lawra Municipality and accessing treatment at the Lawra Municipal Hospital.

The sample size was calculated based on a single population proportion formula, considering 17.0% as the prevalence of dietary diversity among HIV patients with malnutrition as a proxy in determining the sample population [12] at a 95% level of confidence for the two-tail test and a marginal error or level of precision (*d*) = 5%. Thus, the sample size (*n*) then was calculated as follows:

(1) $$\begin{array}{c} N=\frac{1.96^{2}*0.17\left(1-0.83\right)}{0.05^{2}}=216.8. \end{array}$$

The sample size was increased by 25% to account for contingencies such as nonresponse and error recording. The final sample was subsequently adjusted to 270.

The systematic random sampling technique was used to give everyone an equal opportunity of being selected. This technique was used to sample study participants on their antiretroviral therapy clinic days at every K^th^ (2^nd^) interval. *K* was the sampling fraction, which was calculated as *N*/*n* = 1477/270 ≈ 2. Among the first two clients, the starting participant was chosen by lottery as the starting sample. The technique was repeated until the required sample size was achieved.

### 2.3. Data Collection Instrument

A questionnaire that was constructed by the research team and a standard individual dietary diversity tool which was adopted from the standardized FAO's IDDS food questionnaire tool with a 24-hr food recall method [13] were used. This tool was then computerized into an electronic media to aid easy and timely data collection.

### 2.4. Measurement of Independent and Dependent Variables

The main dependent variable was the individual dietary diversity score (low 4 or less food groups and high dietary diversity 5 or more food groups). The primary independent variables included age, sex, marital status, ethnic group, and occupation, opportunistic infection, ART adherence level, cotrimoxazole adherence, herbal drug usage, WHO clinical stages, and nutritional knowledge level. A brief outline of how key independent and outcome variables were measured is as follows.

### 2.5. Measurement of Individual Dietary Diversity Score

The measure of dietary diversity of a household reflects the qualitative process of determining their food consumption and their access to food varieties. Respondents reported the frequency of consumption of each food consumed within the past 24 hours according to the protocols outlined by standardized FAO's IDDS food questionnaire tool and received 1 point if they consumed any food within a particular food group at least once during the last 24 hours prior to the study and 0 point if they never consumed the food (both in and out of home). The 24-hour dietary recall started with breakfast between 6:00 a.m. and 10:00 a.m., followed by lunch (12:00 a.m.–4:00 p.m.) and dinner (8:00 p.m.–12:00 a.m.). In between, these meals were considered as snacks. Based on the 2011 Household Dietary Diversity/Individual Diversity Dietary measuring guidelines of the Food and Agriculture Organization (FAO), the foods eaten by the respondents were classified into 9 food groups: starchy staples (cereals &white roots and tubers); dark green leafy vegetables; other vitamin A rich fruits and vegetables; oils/fats; legumes, nuts, and seeds; other fruits and vegetables; organ meat; milk and milk products; eggs; meat and fish; sea foods.

These groups were then dichotomized into two categories based on the mean Individual Dietary Diversity Score for further analysis of 3.66 ± 0.9. Based on the Food and Agriculture Organization Dietary Diversity Score, rating participants who scored 4 or less food groups were considered as low in dietary diversity while dietary diversity scores greater than or equal to 5 food groups were high [3, 13].

### 2.6. Data Collection Procedures

The data collection tools were programmed into a computer-assisted personal interviewing tool and used for the collection of data. Three data collectors (one HIV/AIDS nurse specialist and two nutrition officers) were recruited and given a one-day training on the survey instruments and the usage of the data collection tools. Data were collected through face-to-face interviews. By evaluating each completed questionnaire daily, the principal investigator ensured that the gathered data were full and consistent, and on-site supervision was carried out throughout data collecting times. Data were collected from December 2020 to February 2021.

### 2.7. Data Quality Control

Previous relevant literature was used to construct the questionnaire [13], which was then adjusted to fit the local situation. Training was provided to enumerators, and pretesting was conducted in a similar ART center in Wa Municipal Hospital using 5% of the sample size prior to the main data collection exercise. Corrections and adjustments were made to the questionnaire as required, following pretesting.

### 2.8. Data Processing and Analysis

Data were exported from the computer-assisted personal interviewing device as an Excel sheet. The data were cleaned, coded, and imported into SPSS version 25 for analysis. The study analysis was done by computing proportions and summary statistics. Then, the information was presented by using a frequency table, graphs, and cross-tabulation. Respondent's dietary diversity was computed by calculating the total number of food groups consumed within the previous 24 hr, dichotomous into 0–4 as low diversity and 5+ as high diversity [3]. Bivariable and multivariable logistic regression analyses were performed to identify factors associated with individual dietary diversity. Each independent variable was entered in the bivariate logistic regression. Finally, variables, which show associations in the bivariable logistic regression at *p* value of less than 0.02 with 95% CI, were entered into multivariable logistic regression and were declared statistically significant at *p* value of less than 0.05.

### 2.9. Ethical Clearance

Ethical clearance was obtained from the Institutional Review Board (IRB) of the Navrongo Health Research Center (ID number NHRCIRB375). Formal permission was also obtained from the Upper West Regional Director of Health Services, the Lawra Municipal Director of Health Services, and the Medical Superintendent of the Hospital. The objectives and relevance of the study were adequately outlined to the study participants. Written consent was obtained from participants before they participated in the study. The confidentiality of information of study participants remains ensured by the researcher. Each participant was assigned a unique code to prevent the documentation of the participant's names.

## 3. Results

### 3.1. Sociodemographic Characteristics of Study Participants

The study included a total of 269 participants representing a response rate of 98.5%. The mean age of the study participants was 43.3 ± 11.0 years while the range of ages was 35–49 years. The majority, 175 (65.1%), of the study participants were females. Christianity, 209 (77.7%), was the dominant religion among the study population, 178 (66.2%) were married, and 193 (71.7%) had no formal education. Out of the total, 242 (90.0%) of study participants had irregular sources of income and 129 (48.0%) were found in the lower wealth quintile (Table 1).

### 3.2. Health-Related and Behavioral Characteristics of Respondents

The majority, 152 (56.5%), of the study participants were on the antiretroviral drugs for 5–10 years. TDR + 3TC + EFV (tenofovir + lamivudine + efavirenz) were the common ART drug among 207 respondents (76.9%). Two hundred and fifty-five (94.8%) of the adults were on cotrimoxazole prophylaxis. The majority, 130 (48.3%), were at WHO clinical stage 3 of the disease with 262 (97.4%) of them reporting no opportunistic infections. Almost all 259 (96.3%) of the adults infected with HIV/AIDS adhered positively to their ART regimen with 264 (98.1%) not experiencing any gastrointestinal symptoms.

Out of the total, 141 (52.4%) of respondents received dietary counseling while receiving treatments. The majority of the counseling was received from a nutritionist in the facility. 209 (77.7%) of the participants had good knowledge on the nutritional requirements while living with HIV/AIDS (Table 2).

### 3.3. Dietary Practices of HIV-/AIDS-Infected Adults on ART Living in Lawra Municipality

**Figure 1:**
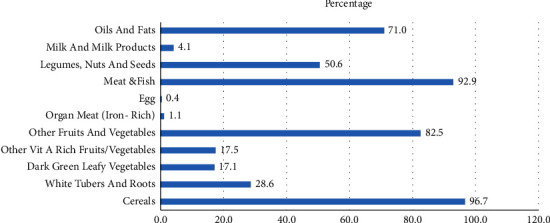
Food groups eaten within 24 hours for HIV-positive adults (18–85 years) attending antiretroviral therapy clinics in Lawra Hospital.

The frequency of consuming specific food groups within the past 24 hours prior to this study was assessed among HIV-/AIDS-infected adults. Starchy staple foods and flesh food were the most commonly consumed foods, while organ meats, dairy products, and eggs were eaten by less than 5% of the respondents (Figure 1). Individual dietary diversity score (IDDS) was used as a proxy measure of the nutritional quality of an individual's diet. The mean dietary diversity score (DDS) of the study population from nine food groups was 3.7 ± 0.9. The proportion of participants not meeting the minimum dietary diversity (<5 food groups) was 233 (86.6%). In terms of frequency of meals, 66.8% of the study participants are three times or more in a day.

### 3.4. Predictors of Dietary Diversity among Adults on Antiretroviral Therapy in Lawra Municipal

Compared to patients who were unemployed, nonfarmer employment was 10.8 times more likely to have a high dietary diversity score, AOR = 10.76 (95% CI: 1.03–112.35). Women aged 18–27 were 5.95 times more likely to have a high dietary diversity score, AOR = 10.76 (95% CI: 1.18–30.07), compared to their counterparts who were more than 48 years old. Adult HIV-positive respondents who were not on cotrimoxazole prophylaxis were 3.8 times more likely to have high dietary diversity, compared to women who were using the medicine AOR = 3.76 (95% CI: 1.02–14.37) (Table 3).

## 4. Discussion

The main aim of the study was to assess the magnitude and associated factors of dietary diversity among adult patients on ART. It was evident from this present study that only 13.4% of HIV-infected adults had a diversified diet 24 hours preceding the survey. Much higher proportions were reported in similar studies in different parts of Ghana (19.3%), Motta Town, Ethiopia (29.5%), and Metema Hospital (32.1%) [4, 14, 15]. This difference in findings might have risen due to the variation in the measurement of the dietary diversity and the variation in cropping seasons of the study locations. This current study was conducted in the lean season (December to February) and might have accounted for the low dietary diversity reported among study participants, as most of their stocks might have been exhausted. The findings of the current study possibly suggest a high risk of malnutrition among persons living with HIV in the Lawra municipality during the lean seasons which is associated with food insecurity. Therefore, interventions targeting nutritional status enhancement must be structured to peak around this season of food insecurity.

The most consumed food group by persons living with HIV/AIDS in Lawra municipality was starch staples and dry fish as well as fats and oils. Similar consumption patterns were reported by Tesfaw et al. [4] in Ethiopia and Sanusi in Butajira, Nigeria [16]. The high consumption of these starch foods especially the grains (maize) might be influenced by several factors including the fact that it is a major staple food in northern Ghana and is readily available in most traditional homes. The high consumption of these high energy-sourcing food groups may provide HIV-infected persons with energy, but their micronutrient requirements such as iron, vitamins, and minerals that are required for the boosting of their immune system will be compromised.

Strong predictors of a positive dietary diversity included the occupation of the individual living with HIV/AIDS. When compared to those who were unemployed, nonfarmer employees were ten times more likely to have a high dietary diversity. That is, HIV-positive individuals who were gainfully employed were more likely to have a diverse diet than HIV-positive adults who were jobless. Similar associations were also reported in studies conducted in Kampala, Uganda, Durgapur, West Bengal, and Mettema Hospital, Ethiopia [17–19]. Their ability to earn an income on a daily or monthly basis offers them an opportunity to have the purchasing power, therefore capable of catering for their dietary requirements.

Also, the study showed that patients who were between the age of 18–27 years were five times more likely to have a high dietary diversity compared to patients who were 48 years and older. Similar findings were found in Durgapur-West Bengal, and Mali [19, 20]. It is, therefore, evident that as adult age increases, there is a reduction in the adequacy of their diet in meeting their nutritional needs.

Patients who were not taking the cotrimoxazole prophylaxis were 4 fold time likely to have a high dietary diversity compared to those who did. This could be due to the side effects of the prophylaxis drug. Similar reports were presented in Mettema, Ethiopia (Woldemariam et al. 2018).

## 5. Conclusion

The findings of this study reveal a high level of low dietary diversity and possible food insecurity among HIV-/AIDS-infected persons living in Lawra Municipality. Employment status, cotrimoxazole prophylaxis adherence, and patients aged 18–27 years were positive predictors of an adequate dietary diversity score. Therefore, interventions targeting household economic status improvement and food security are necessitated in boosting the dietary diversity of antiretroviral treatment patients.

### 5.1. Limitation of the Study

There are some limitations to this study including recall bias and social desirability bias. Furthermore, in applying the FANTA HDDS questionnaire in this study, we did not take seasonality into account.

## Figures and Tables

**Table 1:** Sociodemographic characteristics of study participants.

Variables	Frequency (*N* = 269)	Percentage (%)
Ages		
Under 35 years	57	21.2
35–49 years	144	53.5
At least 50 years	68	25.3
Sex		
Male	94	34.9
Female	175	65.1
Religion		
Christian	209	77.7
Islam	16	5.9
Traditional	27	10
No religion	17	6.3
Marital status		
Single	29	10.8
Married	178	66.2
Cohabitating	10	3.7
Separated/divorced	8	3
Widow	44	16.4
Education		
None	193	71.7
Primary school	28	10.4
JHS/middle school	33	12.3
SHS/secondary school	10	3.7
Higher/tertiary	5	1.9
Occupation		
Farmer	204	75.8
Trader/business/artisan	38	14.1
Unemployed	10	3.7
Government employee	8	3
Private sector employee	6	2.2
Student	3	1.1
Wealth index		
Lowest quintile	129	48
Second quintile	114	42.4
Third quintile	22	8.2
Fourth quintile	4	1.5

JHS: junior high school and SHS: senior high school.

**Table 2:** Health-related and behavioral characteristics of HIV-positive adults receiving antiretroviral therapy.

Variable	Frequency (*n*)	Percentage (%)
Duration on ART (years)		
Under 5	96	35.7
5–10	152	56.5
10^+^	21	7.8
Type of ART drug		
TDF + 3TC + DTG	37	13.8
TDF + FTC + DTG	10	3.7
TDF + 3TC + EFV	207	76.9
TDF + FTC + EFV	11	4.1
TDF + 3TC + NVP	4	1.5
On cotrimoxazole prophylaxis		
Yes	255	94.8
No	14	5.2
WHO clinical stage		
Stage 1	42	15.6
Stage 2	79	29.4
Stage 3	130	48.3
Stage 4	18	6.7
Opportunistic infection (OI)		
No	262	97.4
Yes	7	2.6
Adherence to ART in the past month		
Good	259	96.3
Poor	10	3.7
Experienced any GI symptoms		
Yes	5	1.9
No	264	98.1
Received dietary counseling		
Yes	141	52.4
No	128	47.6
Source of counseling		
Radio	9	6.4
Nutritionist/dietitian	91	64.5
Family/friends	33	23.4
Other health professionals	8	5.7
Nutritional knowledge of respondents		
Poor	60	22.3
Good	209	77.7

**Table 3:** Predictors of dietary diversity among adults on antiretroviral therapy.

Variable	Categories	Classification of IDDS		COR (95% CI)	AOR (95% CI)
		Low *N* (%)	High *N* (%)		
On cotrimoxazole prophylaxis?	Yes	223 (87.5)	32 (12.5)	Reference	Reference
	No	10 (71.4)	4 (28.6)	2.79 (0.83–9.42)	**3.76 (1.02–14.37)** ^*∗*^

Educational level	None	170 (88.1)	23 (11.9)	Reference	Reference
	Low (primary and JHS)	53 (86.9)	8 (13.1)	1.12 (0.47–2.64)	1.15 (0.56–2.74)
	High (at least SHS)	10 (66.7)	5 (33.3)	3.70 (1.16–11.77)^*∗∗*^	2.86 (1.36–12.01)

Occupation status	Unemployed	12 (92.3)	1 (7.7)	Reference	Reference
	Farmer	187 (91.7)	17 (8.3)	1.09 (0.13–8.91)	1.78 (0.18–18.04)
	Nonfarmer employment	34 (65.4)	18 (34.6)	6.35 (0.76–52.85)	**10.76 (1.03–112.35)** ^*∗*^

Age (years)	18–27	8 (66.7)	4 (33.3)	4.33 (1.09–17.30)^*∗*^	**5.95 (1.18–30.07)** ^*∗*^
	28–37	62 (89.9)	7 (10.1)	0.98 (0.35–2.78)	1.17 (0.39–3.55)
	38–47	85 (84.2)	16 (15.8)	1.63 (0.68–3.90)	1.58 (0.63–3.99)
	>48	78 (89.7)	9 (10.3)	Reference	Reference

Bold values represent significance at *p* value <0.05. ^∗^*p* value <0.05. ^∗∗^*p* value <0.01.

## Data Availability

Data will be available upon request from the corresponding authors.

## Abbreviations

HIV: Human immunodeficiency virus
AIDS: Acquired immune deficiency syndrome
ART: Antiretroviral drugs
CI: Confidence intervals
HDDS: Household dietary diversity score.
